# Apoptosis levels in bovine Johne’s disease ileal lesions and association with bacterial numbers

**DOI:** 10.1177/03009858211025790

**Published:** 2021-06-30

**Authors:** Amalia Naranjo- Lucena, Laura Garza-Cuartero, Conor McAloon, Grace Mulcahy, Annetta Zintl, José Perez, Alan Wolfe

**Affiliations:** 1 8797 University College Dublin, Dublin, Ireland; 2 16735 University of Córdoba, Córdoba, Spain

**Keywords:** apoptosis, bovine, FoxP3, granulomatous lesion, Johne’s disease, multinucleated giant cells, epithelioid macrophage, paratuberculosis, T cells

## Abstract

Johne’s disease (JD) is a chronic granulomatous enteritis caused by *Mycobacterium avium* subsp. *paratuberculosis* (MAP). While it is generally accepted that MAP employs immune subversion mechanisms, aspects of the host-pathogen relationship are not fully understood. We sampled 3 ileal tissue sections from 17 naturally infected cattle (*n* = 51 sections) to analyze differences in cell types, apoptosis, and phagocytic cells. Diffuse multibacillary (DM) was the most common lesion type (*n* = 17) followed by diffuse intermediate (DI; *n* = 15). DM lesions had significantly greater proportion of Treg cells (CD3^+^ FoxP3^+)^ relative to all CD3^+^ T cells as compared to DI forms (*P* < .05). CD68^+^ individual cell size was significantly smaller in DM than in diffuse lymphocytic (DL) forms (*P* < .05). Area of caspase-3 positivity (apoptosis) was greater in DM lesions than DL (*P* < .05) and DI (*P* < .0001), and was linked to higher numbers of MAP within the macrophage.

Johne’s disease (JD) is a chronic wasting disease characterized by the development of granulomatous lesions in the intestinal mucosa of ruminants.^19^ Animals with clinical disease develop chronic diarrhea and rapid weight loss. JD represents a challenge to the farming industry due to its impact on animal production during the subclinical phase, which is aggravated by the difficulty of detecting *Mycobacterium avium* subsp. *Paratuberculosis* (MAP)-infected animals at an early stage of infection.^12^

The pathogenesis of JD involves the inability of the host immune response to control the infection. This inability is partly orchestrated by MAP, which induces the host immune response to shift from a protective Th1 to a Th2 or mixed (Th1/Th2) immune response during the course of infection, facilitating the survival of the organisms within the macrophages in the intestinal mucosa.^2,17^ In addition, MAP can subvert the host immune response by reducing macrophage responsiveness to interferon (IFN)-γ, blocking phagolysosome fusion, inducing interleukin (IL)-10 production, inhibiting the recruitment of nitric oxide synthase, and manipulating host cell apoptosis.^1,2,10,11,15^

Histological changes induced by MAP vary in terms of their constituent cell makeup, number of bacilli, and expression of cytokines.^4,7–9^ To further investigate these differences and the immune response to JD, we sourced cattle with clinical signs and explored cell types, phagocyte size, and apoptosis levels associated with each lesion type.

All 17 cattle included in the study had overt clinical signs of JD (ie, severe diarrhea and weight loss). Cows were between 2 and 7 years of age and were sourced from 16 herds. Thirteen were Holstein-Friesian, and 4 were Limousin. Following euthanasia, fecal and tissue samples from the distal, mid, and proximal ileum were collected (taken 20, 40, and 60 cm from the ileocecal valve). JD was confirmed by fecal PCR (polymerase chain reaction) and/or serological ELISA (enzyme-linked immunosorbent assay) and the presence of acid-fast bacteria (AFB) in the ileal mucosa. Animals and procedures carried out were approved for exemption from full ethical review by the UCD Animal Research Committee (AREC-E-16-38-Mulcahy).

Fourteen of 17 cases had moderate to severe gross thickening of the ileal mucosa and moderately enlarged ileocaecal lymph node (ILN). In 3 of these cases, thickening of the mucosa extended into the ileocecal valve and the cecum. In contrast, the remaining 3 cases had minimal gross lesions of the ileal mucosa. Samples were routinely fixed in 10% neutral-buffered formalin, embedded in paraffin, sectioned at 4 μm, and stained with hematoxylin and eosin (HE) and by the Ziehl–Neelsen (ZN) method for AFB.

Tissue lesions were evaluated histologically, and all ileal sections classified as previously described.^8^ Briefly, focal lesions were characterized by small well-demarcated granulomas (including 5 to 30 macrophages) present in the lymphoid tissue of the distal ileum. Multifocal (MF) lesions consisted of multiple granulomas and scattered Langhans giant cells (LGC) present in the lymphoid tissue and in the intestinal lamina propria. Diffuse lesions were further divided into 3 subcategories: (1) multibacillary (DM), where foamy macrophages and epithelioid cells diffusely infiltrated the intestinal wall and associated lymph nodes, and large numbers of mycobacteria were found; (2) lymphocytic (DL) paucibacillary, with lymphocytes as the main inflammatory cell infiltrating the lamina propria, few small granulomas in the Peyer’s patches and submucosa, and few mycobacteria; and (3) intermediate (DI) paucibacillary, with varying numbers of lymphocytes, plasma cells, giant cells and macrophages sometimes forming small granulomas, and with mycobacteria only present in small numbers in the lymph nodes. Additionally, the number of bacteria found in each section was estimated and scored subjectively by 2 researchers independently, including a diplomate of the European College of Veterinary Pathologists, as follows: 0 = No bacteria present; 1 = Bacteria present, but in very low numbers with individual bacilli contained within macrophages; 2 = Moderate number of bacteria with a more widespread presence in the tissue forming clusters within the macrophages but not diffusely within the cytoplasm; and 3 = High numbers of bacteria, widespread within tissue, and occupying most or all the cytoplasm.

Immunohistochemistry (IHC) was carried out using standard methods. Antibodies employed were monoclonal mouse anti-human CD68, polyclonal rabbit anti-human CD3, mouse/rat monoclonal anti-FoxP3, and polyclonal rabbit anti-human caspase 3. Methods for IHC and imaging are summarized in the Supplemental Materials. Caspase 3^+^ staining showed a granular/diffuse cytoplasmic distribution, mainly concentrated in epithelioid cells located within lesions, but not in macrophages outside lesions. Positive area rather than number of positive cells was considered, as the distribution of caspase labeling did not allow for reliable identification of individual cells (Figs. 1 [Fig fig1-03009858211025790] [Fig fig1-03009858211025790]–4).

**Figures 1–4. fig1-03009858211025790:**
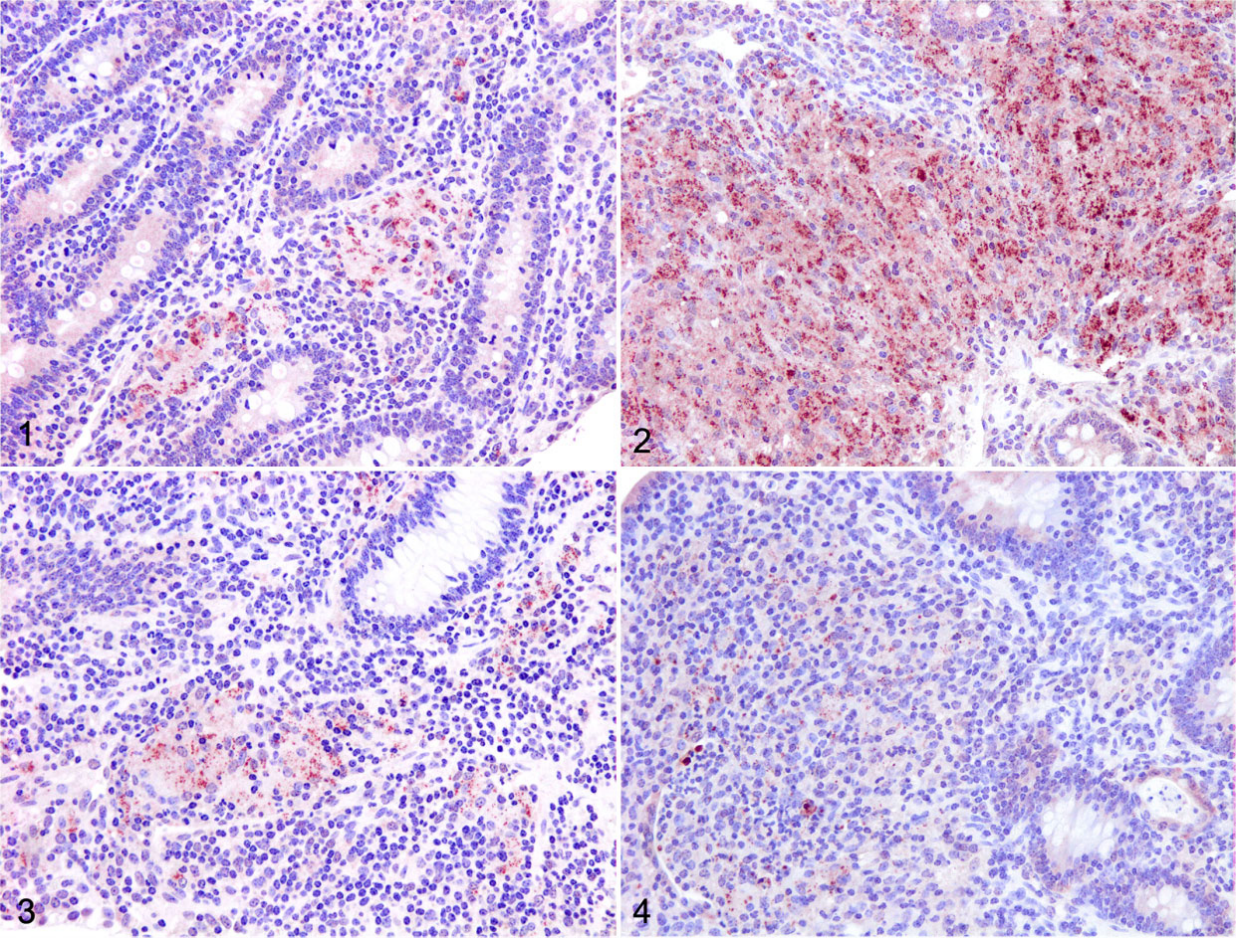
Johne’s disease, ileum, bovine. Immunohistochemistry for caspase 3. **Figure 1.** Case 14. Multifocal lesion. **Figure 2.** Case 2. Diffuse multibacillary lesion. **Figure 3.** Case 13. Diffuse lymphocytic lesion. **Figure 4.** Case 8. Diffuse intermediate lesion.

Statistical analysis was carried out with GraphPad Prism version 7 including data for all sections except for those that did not fit into any classification. Due to the difficulty in sourcing naturally infected animals the number of animals included in the study was low (*n* = 17). As several cases presented with more than one lesion type, data from the 3 sections from each animal were compared individually; however, it is important to stress that these samples were not independent. Where the pathologist gave a score of 1/2 or 2/3 for a particular section, the lower score was assigned for statistical purposes. ANOVA with posttest Tukey’s multiple comparisons was carried out for multiple groups, and Student’s *t*-test when comparing 2 groups. If data were nonnormal or less than 3 individuals showed a certain lesion classification, Kruskal-Wallis with posttest Dunn’s and Mann-Whitney *U* test were employed to compare multiple groups or pairs, respectively. Results were considered significant where *P* < .05.

Histologically, JD is associated with diffuse granulomatous lesions in the ileal mucosa which may be either paucibacillary or multibacillary.^4,8,14^ Animals with multibacillary lesions have more severe clinical disease, and a less effective cell mediated immune (CMI) response than animals with paucibacillary lesions.^3,6^ In this study, 4 different lesion types were encountered in the 17 animals sampled. Overall, there were 17 DM lesions from 9 cattle, 15 DI lesions from 7 cattle, 6 DL lesions from 3 cattle, and 6 MF lesions from 2 cattle (Table 1). DM lesions showed significantly greater mean areas of apoptosis compared to DI (*P* < .0001) and DL (*P* < .05; Fig. 5g). None of the tissue sections from cases 5 and 9, and the distal ileum section from case 6 could be classified according to González et al.^8^ Seven cases presented with more than one lesion category in the ileal sections.

**Table 1.** Lesion classification and acid-fast bacteria (AFB) scores encountered in proximal, mid, and distal ileum from the 17 cattle diagnosed with Johne’s disease.

Case		Proximal Ileum	Mid Ileum	Distal Ileum
1. LM	Lesion type	Multifocal	Multifocal	Multifocal
	AFB score	0	0	0
2. HF	Lesion type	DM	DM	DM
	AFB score	3	3	3
3. HF	Lesion type	DI	DM	DI
	AFB score	2	2	1
4. HF	Lesion type	DI	DI	DI
	AFB score	0	0	0
5. LM	Lesion type	Enteritis	Focal	Enteritis
	AFB score	0	0	0
6. HF	Lesion type	DL	DL	Enteritis
	AFB score	0	0	0
7. HF	Lesion type	DM	DM	DM
	AFB score	2	2	2
8. HF	Lesion type	DI	DI	DM
	AFB score	2	2	2
9. HF	Lesion type	Mild enteritis	Normal	Normal
	AFB score	0	0	0
10. HF	Lesion type	DI	DM	DI
	AFB score	1	1	1
11. HF	Lesion type	DM	DM	DM
	AFB score	3	3	3
12. HF	Lesion type	DM	DM	DM
	AFB score	3	3	3
13. HF	Lesion type	DL	DL	DM
	AFB score	2/3	2/3	3
14. HF	Lesion type	Multifocal	Multifocal	Multifocal
	AFB score	2	2/3	2/3
15. LM	Lesion type	DI	DI	DM
	AFB score	1	1	1/2
16. LM	Lesion type	DL	DL	DI
	AFB score	0	0	0
17. HF	Lesion type	DI	DI	DI
	AFB score	0	1	1

Abbreviations: AFB, acid-fast bacteria; LM, Limousin; HF, Holstein-Friesians; DM, diffuse multibacillary; DL, diffuse lymphocytic; DI, diffuse intermediate; 0 = no bacteria; 1 = bacteria in very low numbers; 2 = moderate number of bacteria; 3 = high numbers of bacteria.

**Figure 5.**
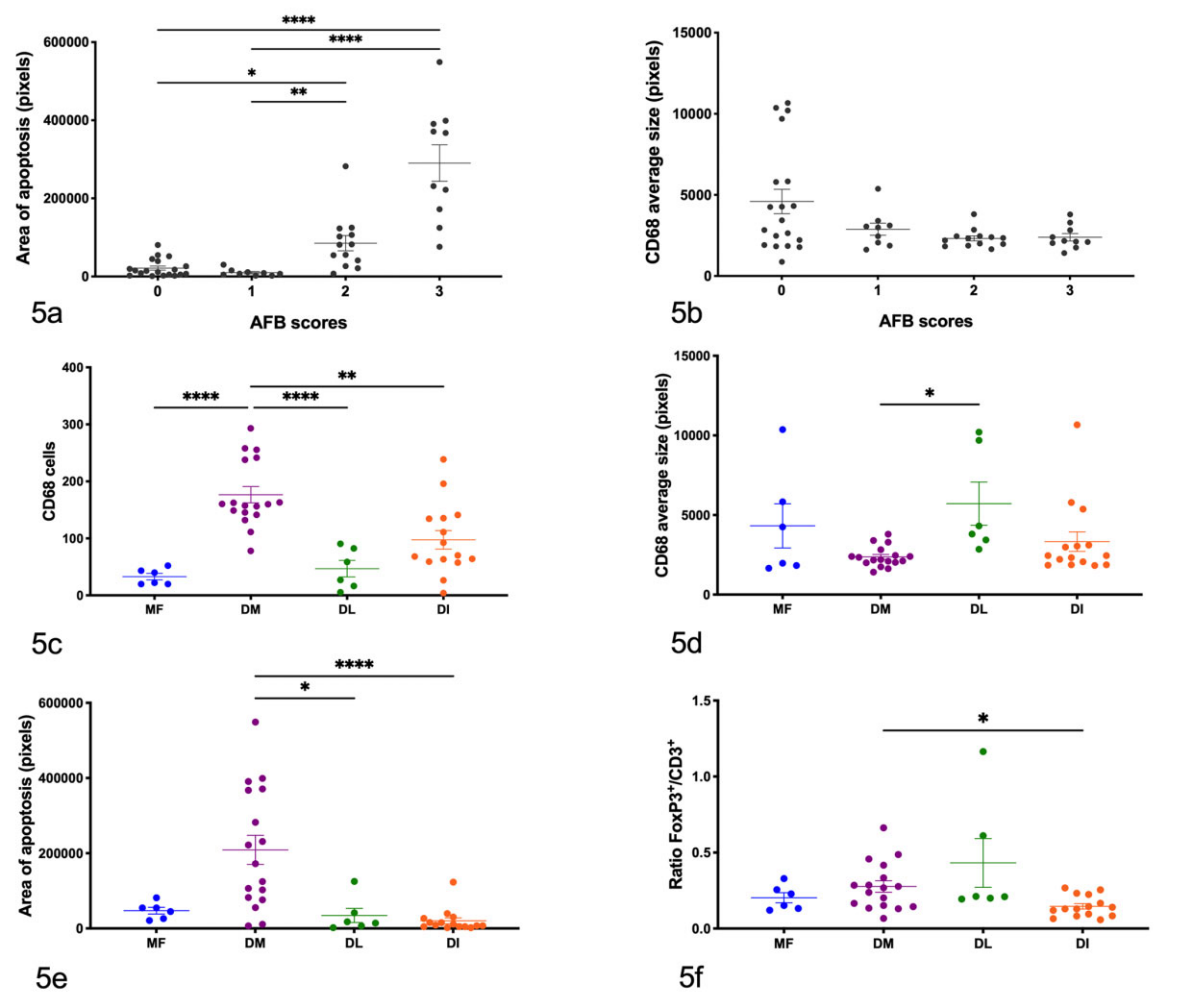
Quantification of apoptosis, number/ratio, and size of cell types based on immunohistochemistry. (a) Mean area of apoptosis (caspase-3^+^) in ileal sections for samples with different acid-fast bacteria (AFB) scores. (b) Mean size of CD68^+^ cells in ileal sections for samples with different AFB scores. (c) Mean number of CD68^+^ cells in different lesion classes. (d) Mean size of CD68^+^ cells in different lesion classes. (e) Mean area of apoptosis (caspase-3^+^) in different lesion classes. (f) Ratio of the number of FoxP3^+^ to CD3^+^ cells in different lesion classes. Tissue sections are represented individually. AFB are scored from 0 to 3. Ileal sections are categorized as multifocal (MF), diffuse multibacillary (DM), diffuse lymphocytic (DL), and diffuse intermediate (DI) lesions. Horizontal lines and error bars show mean ± SEM. ****P < .0001, ***P < .001, **P = .002, *P < .05.

Caspases are specialized proteases that play an essential role in programmed cell death, including apoptosis.^13^ Apoptosis of infected cells can be beneficial to the host by removing pathogens, limiting acute inflammation, and facilitating MAP antigen presentation through efferocytosis (phagocytosis of apoptotic cells) to stimulate adaptive responses. On the other hand, apoptotic cells are also phagocytosed by healthy macrophages, which can be beneficial to the pathogen by helping spread the infection and avoiding the activation of uninfected macrophages. It is not possible to tell whether it is the host or the pathogen that orchestrates the apoptotic process, and both possibilities have been proposed using valid arguments.^2,18^ In our tissue sections, higher AFB scores were associated with greater areas of cleaved activated caspase-3 (*r* = 0.7, *P <* .0001, Spearman’s rank correlation coefficient), indicating that the number of mycobacteria in the tissues were positively associated with a greater area of apoptosis. DM lesions showed a variation in AFB scores and apoptosis areas; however, a positive association within that cohort was also demonstrated (*r* = 0.67, *P* < .0068). The data suggest that macrophages that show increased signs of apoptosis are therefore less able to clear the infection. However, it is possible that other reasons for variation in the apoptotic process are taking place that we cannot identify in this study. Whether cell apoptosis is a strategy employed by MAP to survive within the infected macrophages and replicate still needs to be elucidated.

We observed that the ratio of FoxP3^+^/CD3^+^ T cells was significantly greater in DM lesions than in DI lesions (*P* < .05; Fig. 5f), indicating different T cell population proportions between lesion types. This anti-inflammatory response may be due to a higher antigen load present in DM lesions. However, Tregs would be expected to be more common in DL and intermediate lesions than in the DM type. It has been shown that FoxP3 is highly expressed in ileal tissues from subclinical but not clinical JD cattle, indicating that this cell type may be related to the containment of MAP in the early or latent stages of infection.^5^

As expected, numbers of CD68-positive cells (macrophages/LGCs) were significantly higher in DM lesions than in multifocal (*P* < .0001), DL (*P* < .001), or DI (*P* < .0001) type lesions (Fig. 5c). However, the size of CD68^+^ cells in DM lesions was significantly smaller than in DL ones (*P* = .018; Fig. 5d). Average size of CD68^+^ cells was calculated as an indirect indication of the presence of LGCs. We observed that smaller CD68^+^ cells contained a greater number of bacteria (Fig. 5b), although this trend was not statistically significant. One of the mechanisms used by MAP to facilitate its survival in macrophages is the overexpression of IL-10, and in fact, DM lesions have been associated with alternatively activated macrophages (M2) (which secrete anti-inflammatory molecules like IL-10).^7,11^ Shrivastava and Bagchi showed that peripheral blood mononuclear cells from human tuberculosis patients produced increased levels of IL-10 after Concanavalin A or purified protein derivative stimulation, and exogenous addition of IL-10 to monocytes in vitro resulted in a reduced formation of multinucleated giant cells.^16^ It is possible that MAP also limits the fusion of new monocytes to form LGCs through this process.

The immune response to MAP developed by the host is still not fully understood. In our small group of naturally infected animals, DM lesions were the most common, followed by DI. However, the CD68^+^ cells present in DM lesions were smaller. No significant differences were observed between age groups and breeds (data not shown). To our knowledge, this is the first time that caspase-3 in tissue sections of MAP-infected animals has been quantified and directly linked to higher numbers of bacteria in situ. These results pose the questions as to what extent MAP can manipulate the activity of phagocytes, and ultimately the host immune response.

## Supplemental Material

Supplemental Material, sj-pdf-1-vet-10.1177_03009858211025790 - Apoptosis levels in bovine Johne’s disease ileal lesions and association with bacterial numbers Click here for additional data file. Supplemental Material, sj-pdf-1-vet-10.1177_03009858211025790 for Apoptosis levels in bovine Johne’s disease ileal lesions and association with bacterial numbers by Amalia Naranjo- Lucena, Laura Garza-Cuartero, Conor McAloon, Grace Mulcahy, Annetta Zintl, José Perez and Alan Wolfe in Veterinary Pathology
