# Supplementary material for: Apoptosis levels in bovine Johne’s disease ileal lesions and association with bacterial numbers
Source: Vet Pathol. 2021 Jun 30;58(6):1086–90. doi: 10.1177/03009858211025790 (PMC8581713; doi:10.1177/03009858211025790)
Supplement: Supplemental Material, sj-pdf-1-vet-10.1177_03009858211025790 - Apoptosis levels in bovine Johne’s disease ileal lesions and association with bacterial numbers [file sj-pdf-1-vet-10.1177_03009858211025790.pdf]

Immunohistochemistry methods

Immunohistochemistry (IHC) analysis was carried out using the avidin-biotin-peroxidase method. Antibodies used are shown in Table S1. Initially CD163 and iNOS were also included in the panel in order to determine macrophage phenotype. However, unfortunately, the optimisation of CD163 and iNOS was not successful.

For IHC using CD68, CD3, and Caspase 3, slides were first immersed in xylene (5 min), followed by 100 % alcohol (10 min). Subsequently, endogenous peroxidase was blocked by incubating slides in 3% H_2O_2 for 30 min in the dark. Hydration was then continued by sequentially immersing slides in 96 % alcohol (5 min), 70 % alcohol (5 min) and water (5 min). Next, enzymatic treatment was performed by incubating the slides in either 0.325% protease (Sigma-Aldrich) for 10 min while agitating, or in citrate buffer (Recipe below) (Table 1) for 4 min at 800 W in the microwave oven, followed by 6 min at 90 W. Slides were left to cool down to 45 ºC with the sample container inside a water bath and rinsed thoroughly with three changes of Phosphate-buffered saline (PBS) for 10 min. Background binding was blocked by incubating the slides with 100 µL of 10 % normal goat serum (NGS) in PBS for 30 min at room temperature (RT). Following removal of the NGS, 100 µL of the various primary antibodies (diluted in 10% NGS/PBS) were added and slides incubated overnight at 4 ºC.

The IHC protocol for FoxP3 was slightly different. Rehydration was performed and followed immediately by an incubation in citrate solution for 10 min at 121 ºC in the autoclave. After cooling down to 45 ºC, three washes in PBST80 (PBS + 0.5% Tween 80, PBST80) for 5 min followed by peroxidase blocking for 20 min in 1 % H_2O_2 (in PBST80) was performed. FoxP3 samples were then washed twice for 5 min in PBST80, and once in PBS. Background blocking was performed by incubating for 1 hour in NGS 25% in PBST80. Then, the excess of NGS was removed and 100 µL of FoxP3 antibody (diluted in 10% NGS/PBST80) were added, and slides were incubated overnight at 4 ºC.

For all reactions, specific primary antibodies were substituted with PBS diluted non-immune isotype-matched sera as negative control. A previously known positive section was used as positive control.

Supplemental Table S1: Antibodies used for Immunohistochemistry

Target molecule/ receptor	Target cell/ cell process	Antibody details	Enzymatic treatment	Dilution	Secondary antibody
CD68	Macrophages<br>Langhans giant Cells (LGC)	Dako, monoclonal mouse anti-human CD68, clone EBM11	Protease	1:50	Dako, polyclonal goat anti-mouse immunoglobulins biotinylated (1:50)
CD3	T lymphocytes	Dako, polyclonal rabbit Anti-human CD3	Protease	1:200	Dako, polyclonal goat anti-rabbit immunoglobulins biotinylated (1:200)
FoxP3	T regulatory lymphocytes	eBioscience mouse/rat monoclonal anti-FoxP3, clone FJK-16s	Citrate in autoclave	1:100	Vector Laboratories, polyclonal goat anti-rat IgG (H+L) biotinylated (1:100)
Caspase 3	Apoptosis	Diagnostic Biosystems, polyclonal rabbit anti-human caspase 3 RP096	Citrate in microwave	1:500 (Ileum)<br>1:200 (Lymph node)	Dako, polyclonal goat anti-rabbit immunoglobulins biotinylated (1:200)

The following day, slides incubated with each antibody were equilibrated to RT for 1 hour and rinsed three times in PBST80. They were then incubated with 100 µl of the secondary antibody made up in 10% NGS/PBS (Table 1) for 30 min at room temp. Following three washes in PBS (5 min each wash), 100 µL Avidin-Biotin-Peroxidase Complex (Vectastain ABC Kit, Vector Laboratories) were then added and the slides incubated for 1 hour at RT in the dark. They were then washed three times in TRIS buffer (Recipe below) (5 min each wash) and 100 µL of Vector NovaRED (Vector Laboratories) were added. Following incubation for 2 to 5 min and thorough rinsing in tab water for 10 min, the slides were counterstained with 10% haematoxylin (15 seconds), dehydrated by immersion in 70%, 96% and 100% alcohol (1 min each), and 2 min in xylene. Slides were then mounted using Eukitt® Quick-hardening mounting medium (Sigma-Aldrich) and left to air dry at room temperature overnight.

Citrate buffer:
2.96 g/L $C_6H_8O_7$ (Sigma-Aldrich)
Final pH 6
All made up in PBS

TRIS buffer stock:
121.14 g/L Trizma base (Sigma-Aldrich)
Final pH 7.6
All made up in distilled Water

TRIS buffer:
8.5 g/L NaCl (Sigma-Aldrich)
10% TRIS buffer stock
Final pH 7.6
All made up in distilled Water

Imaging methods

Five images per slide at sites where lesions were present were captured (x200, 700 x 600 μm fields) with an Olympus BX43 microscope and an Olympus XC50 camera. Each section was divided into nine squares (3 columns and 3 rows). Images were taken in all four corners and the centre. Cell quantification and image analysis were performed using Image J software (US National Institutes of Health, Bethesda, Maryland). The number and area in pixels of immunolabelled cells was quantified, and a mean of these values for all five images calculated.

The watershed algorithm was employed to split clustered objects by calculating the Euclidian distance map (EDM) and identifying the ultimate eroded points (UEPs). UEPs are then dilated either until the edge of the particle is reached, or the edge touches a region of another (growing) UEP. (https://imagej.nih.gov/ij/docs/guide/146-29.html#sub:Watershed).
